# The College Scale of Minimum Salaries

**Published:** 1921-01-15

**Authors:** 


### THE COLLEGE SCALE OF MINIMUM SALARIES.

### £18 to £500.

The following is the scale of minimum salaries recommended by the College of Nursing, which it is sending to all institutions and other bodies employing nurses.

| Average Number of Beds in daily occupation. | Matron. (Annual increment, £25.) | Assistant Matron. (Annual increment, £15.) | Home Sister. (Annual increment, £10.) | Night Superintendent. (Annual increment, £10.) | Ward Sister. (Annual increment, £5.) | Staff Nurses. After termination of agreement. (Annual increment, £5.) |
|---|---|---|---|---|---|---|
| | £ | £ | £ | £ | £ | £ |
| Above 500 .................. | 500 | 150—255 | 120—160 | 120—150 | 85—120 | 60—70 |
| 300—500 .................. | 400 | 140—245 | 110—150 | do. | do. | do. |
| 200—300 .................. | 350 | 120—225 | 100—140 | do. | do. | do. |
| 100—200 .................. | 300 | 100—160 | do. | 100—140 | do. | do. |
| 50—100 .................. | 200 | 85—130 | — | 85—125 | 80—90 | do. |
| 25— 50 .................. | 200 | do. | — | do. | do. | do. |
| Under 25 .................. | 150 | — | — | — | do. | do. |

Probationers.—1st Year, £18.
2nd Year, £22.
3rd Year, £30.
4th Year, £40.

Sister Tutors.—£150 (annual increment, £15) to £255, if certificated King's College for Women, or other approved College.
£120 (annual increment, £15) to £225, if not holding a Special Certificate.

Non-Resident Posts.—Minimum £250 for fully certificated Nurse, £10 extra for every required special qualification.

Private Nurses.—Fully certificated, 3½ guineas per week.

Resident District Nurses.—£85 to £120.

In every case uniform to be provided, or monetary equivalent.

*December* 1920.

